# Treatment and survival of patients with esophageal and gastric cancer in the Netherlands and Belgium: A population‐based comparison

**DOI:** 10.1002/ijc.70225

**Published:** 2025-11-19

**Authors:** Benthe H. Doeve, Steven C. Kuijper, Geert Silversmit, Lien van Walle, Philippe Nafteux, Camiel Rosman, Pauline A. J. Vissers, Paul Jeene, Laurens V. Beerepoot, Sarah Derks, Amine Karimi, Maarten F. Bijlsma, Hanneke W. M. van Laarhoven, Rob H. A. Verhoeven

**Affiliations:** ^1^ Department of Medical Oncology Amsterdam UMC location University of Amsterdam Amsterdam The Netherlands; ^2^ Cancer Center Amsterdam Cancer Treatment and Quality of Life Amsterdam The Netherlands; ^3^ Laboratory of Experimental Oncology and Radiobiology, Cancer Center Amsterdam Amsterdam UMC and University of Amsterdam Amsterdam The Netherlands; ^4^ Oncode Institute Amsterdam The Netherlands; ^5^ Department of Research & Development Netherlands Comprehensive Cancer Organisation (IKNL) Utrecht The Netherlands; ^6^ Belgian Cancer Registry Brussels Belgium; ^7^ Department of Surgery University Hospitals Leuven Leuven Belgium; ^8^ Department of Surgery Radboud University Medical Center Nijmegen The Netherlands; ^9^ Department of Radiation Oncology Radiotherapiegroep Deventer The Netherlands; ^10^ Department of Medical Oncology Elisabeth Tweesteden Ziekenhuis Tilburg The Netherlands; ^11^ Department of Medical Oncology Amsterdam UMC, Free University Amsterdam The Netherlands; ^12^ Department of Surgery Jessa Ziekenhuis Hasselt Belgium

**Keywords:** Belgium, esophageal cancer, gastric cancer, Netherlands, survival

## Abstract

Despite demographic and socio‐economic similarities between the Netherlands and Belgium, differences in survival for patients with esophageal and gastric cancer between the two countries exist. This population‐based study investigated these survival differences with the aim of identifying explaining factors. We analyzed data from the Netherlands Cancer Registry (*N* = 26,980) and the Belgian Cancer Registry (*N* = 15,097) for patients diagnosed with esophageal or gastric cancer between 2010 and 2016. Survival differences were examined using relative survival, stratified for tumor location and stage. Factors that potentially mediated the survival differences were tested via multivariable excess hazard models controlling for baseline population mortalities. Five‐year relative survival probability was significantly lower for patients from the Netherlands with esophageal cancer (EC) or gastric cancer (GC) (EC: 21% (20%–22%), GC: 20% (19%–21%)) compared to Belgian patients (EC: 24% (23%–25%), GC: 27% (25%–29%)). Across tumor stages, relative survival of patients with esophageal and gastric cancer was lower among patients from the Netherlands. Multivariable excess hazard modeling showed that survival differences of patients with stage IV disease disappeared when adjusting for treatment. This was not observed among patients with stage I–III gastric cancer, where survival differences remained after adjustment for treatment. Survival of patients with esophageal cancer and gastric cancer is generally higher in Belgium. For patients with stage IV disease, this difference can most likely be attributed to differences in treatment. Although treatment explains part of the survival differences for patients with curable disease, other causes remain largely unknown.

## Graphical abstract

What's new?

Marked differences in esophageal and gastric cancer survival rates exist between the Netherlands and Belgium, for reasons that remain unclear. In this study, the authors in‐vestigated these differences and attempted to identify contributing factors. Analyses confirm that relative survival for esophageal cancer and gastric cancer was lower for patients in the Netherlands. Treatment variables partly mediated survival differences for stage I and II esophageal cancer and stage III gastric cancer. By comparison, for stage IV disease, survival differences could be attributed primarily to treatment variables. The findings suggest that cautious approaches to systemic therapy in the Netherlands are potentially restrictive.

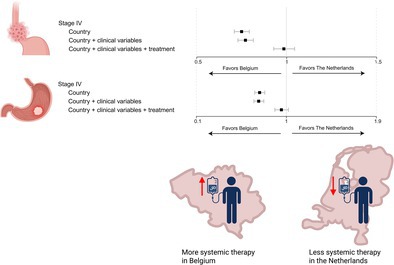

**Abbreviations**
95% CI: 95% confidence interval
AC: adenocarcinoma
BCR: Belgian Cancer Registry
BE: Belgium
CRT: chemoradiotherapy
EC: esophageal cancer
GC: gastric cancer
GP: general practitioner
HR: hazard ratio
IKNL: Netherlands Comprehensive Cancer Organization
IMA: Intermutualistic Agency
NCR: Netherlands Cancer Registry
nCRT: neoadjuvant chemoradiotherapy
NL: Netherlands
RS: relative survival
SCC: squamous cell carcinoma
UICC: International Union Against Cancer

## 1 INTRODUCTION

Survival of patients diagnosed with esophageal and gastric cancer is highly heterogeneous. In patients with non‐metastatic gastroesophageal cancer, the median survival time is around 2 years in the Netherlands and varies depending on tumor‐intrinsic factors, clinical characteristics such as performance status, and treatment.^1^ For patients with metastatic disease, the median survival time is around 4 months but can improve to 1.5 years depending on tumor‐intrinsic factors, performance status and treatment.^2^ In addition to the large heterogeneity of survival among patients, the EUROCARE‐5 study demonstrated that there is also a large international variability in survival. Five‐year age‐standardized relative survival ranged from 5.7% in Lithuania to 21.8% in Belgium for patients with esophageal cancer and from 11.9% in Bulgaria to 34.5% in Iceland for patients with gastric cancer. In the Netherlands, relative survival of patients with esophageal cancer was 8.8 percentage points lower than in Belgium. Similarly, the 5‐year age‐standardized relative survival for gastric cancer patients was 30.5% in Belgium compared to 20.4% in the Netherlands.^3^

A study by Claassen et al.^4^ also found relatively superior survival of patients with metastasized gastric cancer in Belgium compared to the Netherlands. In non‐metastasized gastric cancer, median overall survival was 18 months in the Netherlands compared to 28 months in Belgium.^5^ Interestingly, the Netherlands and Belgium are two western European countries that are similar in a number of demographic characteristics such as socio‐economic status,^6^ tobacco consumption,^7^ obesity and diet.^8^ The observed differences in survival can potentially be explained by several other factors, such as differences in the treatment received, histology, genetics, or tumor stages between the countries.^3^ Currently, it is unknown which factors contribute to the observed differences in survival between the Netherlands and Belgium.

This study aimed to compare survival using real‐world, population‐based, individual‐level data of patients with esophageal or gastric cancer diagnosed and treated in the Netherlands and Belgium, and to identify key factors that could explain differences in survival.

## 2 METHODS

### 2.1 Patients

Patients diagnosed with gastric, gastro‐esophageal junction, and esophageal carcinoma between 2010 and 2016 in the Netherlands and Belgium were identified from the Netherlands Cancer Registry (NCR) and the Belgium Cancer Registry (BCR) respectively. In total, 26,980 individual patients were identified from the NCR and 15,097 individual patients were identified in the BCR.

The NCR is a nationwide database that covers the total population of 17 million in 2016 and includes all patients diagnosed with cancer. Patient identification relies on notifications from the Netherlands' national network and registry of histopathology and cytopathology (PALGA).^9^ Additionally, any non‐pathological verified tumors were included via the Dutch Hospital Database. Trained data managers of the Netherlands Comprehensive Cancer Organization (IKNL) regularly extract information on diagnosis, tumor stage, and treatment directly from each patient's electronic medical records and add this to the NCR.

The BCR is also a nationwide cancer registry, covering the total population of 11.4 million. Data managers hired by hospitals extract the required data from the medical files and routinely report every new diagnosis to the BCR. Independently, the pathology laboratories supply the BCR with their data via the pathology network. Patients with cancer are registered with their unique national Social Security Identification Number, which enables linkage with vital status and date of death, as well as linkage with administrative databases such as the Intermutualistic Agency (IMA) to obtain details on reimbursed diagnostic/therapeutic procedures and pharmaceuticals. IMA data are not directly linked to a specific diagnosis; therefore specific timeframes around the diagnosis date are applied to consider the reimbursed treatment as treatment for the cancer.

### 2.2 Coding and classifications

Primary tumor location was coded as either stomach (C16.1–C16.9) or esophagus (including the gastro‐esophageal junction; international classification of diseases for oncology: C15.0–C16.0). Both adenocarcinomas and squamous cell carcinomas were included as well as carcinoma not otherwise specified. Carcinoma in situ was excluded for this study. Tumors were staged using the International Union Against Cancer (UICC) TNM classification 7th edition. Simplified clinical tumor stages (I, II, III, IV, X) were used in all analyses and were used in addition to tumor location as a stratifying factor. For patients with gastric cancer, a cT4 tumor without specification of a and b, was coded as a cT4a tumor.

For patients with esophageal cancer, treatment was mutually exclusively classified as endoscopic resection, surgical resection only, (neo)adjuvant chemotherapy with surgery, neoadjuvant chemoradiotherapy followed by resection with(out) adjuvant therapy, chemoradiotherapy not followed by resection, systemic therapy only, radiotherapy only and other or unknown. For patients with gastric cancer, treatment was classified as endoscopic resection, surgery only, (neo)adjuvant chemotherapy with surgery, systemic therapy only and other or unknown. Classification was decided on the most invasive treatment. For example, if patients both underwent endoscopic resection and surgical resection, treatment was classified as surgical resection. The category “Other or unknown” included patients that could not be categorized in one of the aforementioned treatments, including best supportive care.

### 2.3 Statistical analysis

Differences in treatment between the Netherlands and Belgium were visualized in bar plots per tumor stage and were interpreted based on visual inspection. We included a category of unknown treatment since any treatment, including best supportive care or a treatment undefinable in our databases, could impact survival. We estimated 5‐year survival using a relative survival framework.^10^ This method adjusts for the baseline population mortality conditional on sex, year of birth, and country. Life tables for the relevant population demographics were used to control for these factors. Differences between relative survival curves of countries were statistically tested with a log‐rank‐type test.^11^ We opted to include all patients despite missing data to limit selection bias and to increase the representativeness of these real‐world data, particularly given the difference in the proportion of missing data between both countries and a previous study that showed a difference in survival in a cohort of only known stages compared to the cohort that included all patients.^5^ In addition, including all patients despite missing variables helped to estimate the parameters for the other covariates, like age.

We further investigated differences in relative survival between the Netherlands and Belgium through excess hazard modeling. Excess hazard modeling can be used to compare the impact of a specific disease on survival between two countries by quantifying the additional risk of death attributable to the disease in each country, controlling for the baseline risk of mortality in the general populations.^12^ This allowed us to estimate the extent to which the survival differences could be explained by demographic, clinical, and treatment variables while controlling for baseline population mortality. An excess hazard ratio of 0.89 in favor of Belgium indicates that the excess mortality rate was 11% lower in Belgium compared to the Netherlands.

Flexible parametric models were applied to model the excess hazard up to 8 years since diagnosis, using the R package mexhaz.^11^ B‐splines were used to specify the baseline excess hazard curve. In a first step, an appropriate baseline excess hazard function was constructed by optimizing the parameters for the B‐spline (spline degree, number of spline knots). The flexible curve was visually compared to a step function, in order to exclude overfitting or a too flexible function. This was performed for the total population, stratified by tumor sublocation and stratified by tumor stages.

Three different excess hazard models were fitted to the data to determine factors which mediated survival differences between countries. The first model only contained country as a predictor variable in the model. The second model contained country and clinical variables (sex, age, and for esophageal cancer also histological subtype), and the third model included country, clinical variables, and treatment (categories as specified in section “*Coding and classifications*”). Age was modeled as a non‐proportional effect. The excess hazard model estimated excess hazard ratios for all the parameters. By sequentially fitting these three models and testing the respective excess hazard ratios for significance, we could observe which factor had the largest impact on the difference in relative survival between the Netherlands and Belgium. Forest plots were created to visualize the excess hazard ratios.

All reported confidence intervals were two‐sided 95% confidence intervals and all *p*‐values <.05 were considered statistically significant. All analyses were performed in R version 4.2.2.^13^

## 3 RESULTS

### 3.1 Baseline characteristics

Baseline characteristics per disease and country are listed in Table 1. There were fewer patients from the Netherlands with esophageal cancer under 59 years of age and there were relatively fewer patients from the Netherlands with gastric cancer that were over 80 years of age. The proportion of squamous cell carcinomas of the esophagus was considerably lower in the Netherlands (NL: 25.0% vs. BE: 37.5%). The number of patients with a T1 tumor was significantly lower in the Netherlands compared to Belgium (esophageal cancer: NL: 4.4% vs. BE: 7.6%, gastric cancer: NL: 3.7% vs. BE: 5.8%) (Table 1) and the number of patients with T1N0 esophageal cancer was also lower (NL: 27%; BE: 50%) whilst the proportion of patients with T2N0 was higher (NL: 73%; BE: 50%) (Supplementary Table 1). In addition, the number of patients with an unknown cT, cN, and cM was significantly lower in the Netherlands. The difference was particularly large for cM; 0.3% of patients with esophageal cancer and 0.8% of patients with gastric cancer in the Netherlands had an unknown cM‐stage compared to 32.5% and 41.9% in Belgium.

**TABLE 1:** Baseline characteristics of all included patients in the Netherlands and Belgium according to tumor location.

	Esophagus		Stomach	
	Netherlands	Belgium	Netherlands	Belgium
*N*	18,294	9617	8686	5480
Sex (%)				
Male	13,652 (74.6)	7206 (74.9)	5258 (60.5)	3183 (58.1)
Female	4642 (25.4)	2411 (25.1)	3428 (39.5)	2297 (41.9)
Age (%)				
<=59	3742 (20.5)	2315 (24.1)	1497 (17.2)	910 (16.6)
60–79	11,445 (62.6)	5481 (57.0)	4874 (56.1)	2593 (47.3)
>=80	3107 (17.0)	1821 (18.9)	2315 (26.7)	1977 (36.1)
Histology (%)				
Adenocarcinoma	13,062 (71.4)	5818 (60.5)	8363 (96.3)	5344 (97.5)
Squamous cell	4567 (25.0)	3603 (37.5)	323 (3.7)	136 (2.5)
Other	665 (3.6)	196 (2.0)	‐	‐
cT (%)				
1	799 (4.4)	727 (7.6)	321 (3.7)	318 (5.8)
2	4425 (24.2)	1192 (12.4)	2144 (24.7)	527 (9.6)
3	6759 (36.9)	3568 (37.1)	1293 (14.9)	1223 (22.3)
4	1216 (6.6)	522 (5.4)	‐	‐
4a	‐	‐	303 (3.5)	344 (6.3)
4b	‐	‐	794 (9.1)	55 (1.0)
*x*	5095 (27.9)	3608 (37.5)	3831 (44.1)	3013 (55.0)
cN (%)				
0	5246 (28.7)	1776 (18.5)	3758 (43.3)	1165 (21.3)
1	6031 (33.0)	2906 (30.2)	1772 (20.4)	992 (18.1)
2	4122 (22.5)	1061 (11.0)	1309 (15.1)	371 (6.8)
3	935 (5.1)	363 (3.8)	172 (2.0)	145 (2.6)
*x*	1960 (10.7)	3511 (36.5)	1675 (19.3)	2807 (51.2)
cM (%)				
0	11,740 (64.2)	4414 (45.9)	5128 (59.0)	1948 (35.5)
1	6495 (35.5)	2081 (21.6)	3491 (40.2)	1237 (22.6)
*x*	59 (0.3)	3122 (32.5)	67 (0.8)	2295 (41.9)

### 3.2 Treatment

**FIGURE 1:**
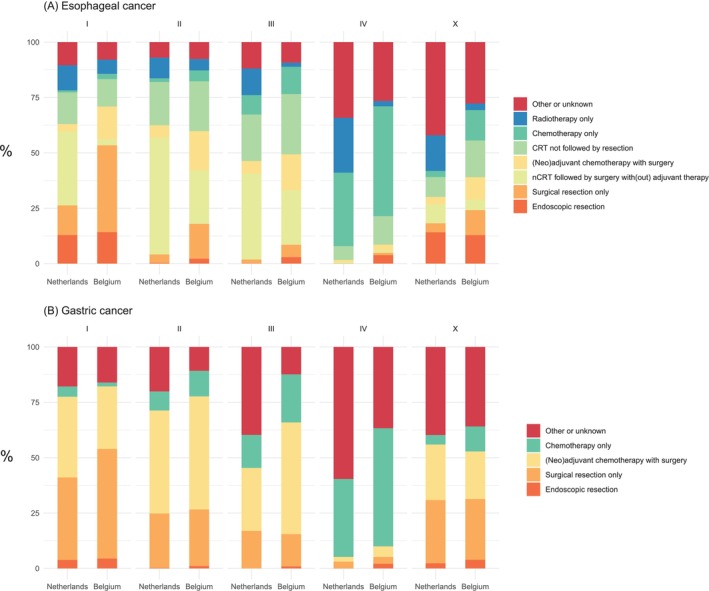
Distribution of treatment categories per clinical stage for patients (A) esophageal cancer and (B) gastric cancer in the Netherlands and Belgium. CRT, chemoradiotherapy; nCRT, neoadjuvant chemoradiotherapy.

There were notable differences in the distribution of types of treatment. For patients with esophageal cancer in the Netherlands, fewer patients received surgery only compared to patients in Belgium with stage I (NL: 13%; BE: 39%), II (NL: 4%; BE: 16%), III (NL: 2%; BE: 6%), IV (NL: 0.1%; BE: 1%) and X (NL: 4%; BE:11%). In stage I, a lower percentage of patients with esophageal cancer was treated with (neo)adjuvant chemotherapy with surgery in the Netherlands (NL: 3%; BE: 15%). Concurrently, there was a higher proportion of patients who received neoadjuvant chemoradiotherapy followed by surgery with(out) adjuvant therapy in clinical stage I (NL: 33%; BE: 3%), II (NL: 53%; BE: 24%), and III (NL: 39%; BE: 25%) compared with Belgium (Figure 1A, Supplementary Table 2). The same trend could be observed in patients with stage I gastric cancer; there was a lower frequency of surgical resection only in the Netherlands compared to Belgium (NL: 37; BE: 50%) and a higher frequency of (neo)adjuvant chemotherapy with surgery (NL: 36%; BE: 28%) (Figure 1B, Supplementary Table 3). On the other hand, there was a lower frequency of (neo)adjuvant chemotherapy with surgery in the Netherlands compared to Belgium in gastric cancer stage II (NL: 47%; BE: 51%) and stage III (NL: 28%; BE: 50%). There was a concurrent increase in the patients treated with other or unknown treatment in the Netherlands (stage II: NL: 20%; BE: 11%, stage III: NL: 40%; BE: 12%).

Patients with stage IV disease in the Netherlands received less systemic therapy only compared to patients in Belgium (esophageal cancer: NL: 33%; BE: 50%, gastric cancer: NL 35%; BE: 53%). Patients with stage IV esophageal and gastric cancer received less (neo)adjuvant chemotherapy with surgery (esophageal cancer: NL: 1%; BE: 3%, gastric cancer: NL 2%; BE 5%). Patients with stage IV esophageal cancer in the Netherlands received less chemoradiotherapy not followed by resection (NL: 6%; BE: 13%). Concurrently, patients from the Netherlands with stage IV gastric cancer received more Other or unknown treatment (gastric cancer: NL: 60% vs. BE: 37%), whilst patients in the Netherlands with stage IV esophageal cancer more often received only radiotherapy (NL: 25%; BE: 2%) or Other or unknown treatment (NL: 34%; BE: 26%). In Supplementary Figure 1, we show that in the Netherlands the latter treatment category largely consists of Best Supportive Care.

### 3.3 Relative survival

**FIGURE 2:**
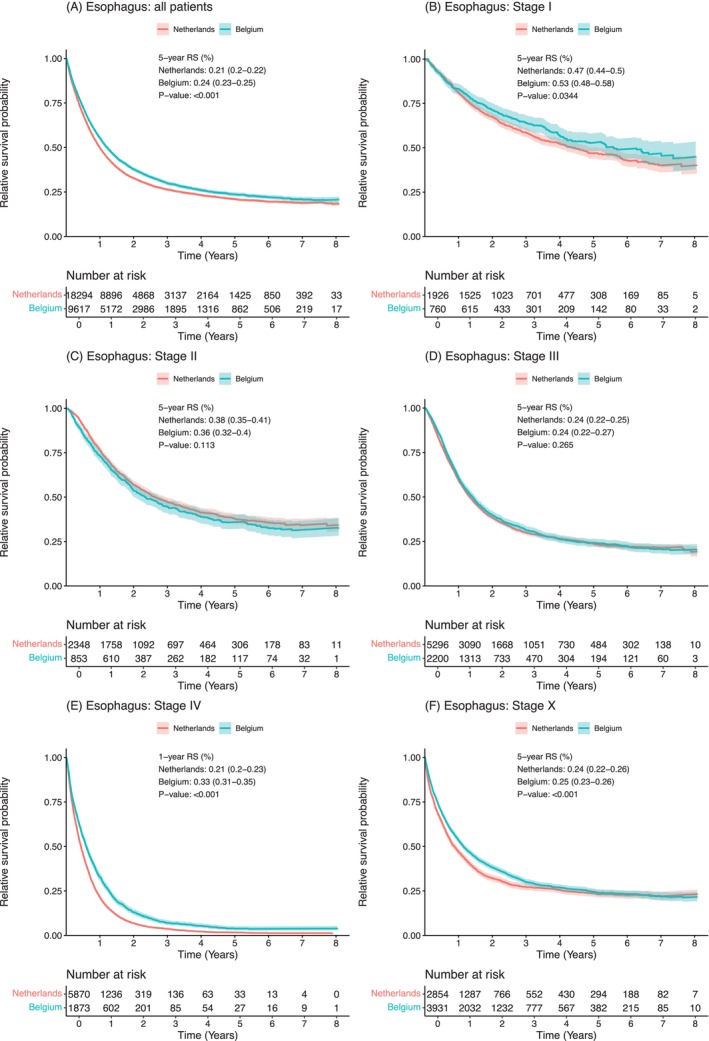
Relative survival probability curves for all patients with esophageal cancer (A), and for patients with stage I (B), stage II (C), stage III (D), stage IV (E), and stage X (F). Relative survival was adjusted for baseline population mortality conditional on sex, year of birth, and country. *p*‐value denotes a log‐rank‐type test.

Five‐year relative survival of all patients with esophageal cancer was significantly lower in the Netherlands (21%, 95% CI: (20%–22%)) compared to Belgium (24%, 95% CI: (23%–25%); *p* <.001) (Figure 2A). Among patients in stage I (5‐year RS: NL: 47% (44%–50%); BE: 53% (48%–58%); *p* = .03), stage IV (1‐year RS: NL: 21% (20%–23%); BE: 33% (31%–35%); *p* <.001) and stage X (5‐year RS: NL: 24% (22%–26%); BE: 25% (23%–26%); *p* <.001) relative survival of patients from the Netherlands was also significantly lower compared to patients from Belgium (Figure 2B,E,F). Among patients in stage II and stage III, no significant differences in 5‐year relative survival were observed (Figure 2C,D). Since survival in esophageal cancer depends on the histological subtype, we performed sensitivity analyses investigating patients with esophageal adenocarcinoma (Supplementary Figure 2) and patients with esophageal squamous cell carcinoma (Supplementary Figure 3) separately. Similar to all patients with esophageal cancer, relative survival was lower for patients in the Netherlands with esophageal adenocarcinoma stage I (5‐year RS: NL: 50% (48%–54%); BE: 56% (50%–63%); *p* = 0.0597) and stage IV (1‐year RS: NL: 22% (20%–23%); BE: 36% (34%–39%); *p* <.001) (Supplementary Figure 2B, E). Relative survival was also lower for patients in the Netherlands with esophageal squamous cell carcinoma stage I (NL: 37% (32%–44%); BE: 48% (40%–57%); *p* = .0502) and stage IV (NL: 24% (21%–26%); BE: 28% (24%–32%); *p* = .00125) (Supplementary Figure 3B, E). Although patients with esophageal squamous cell carcinoma stage X had a lower relative survival in the Netherlands (NL: 13% (10%–17%); BE: 20% (18%–23%); *p* <.001) (Supplementary Figure 3F), this was not the case for patients with esophageal adenocarcinoma stage X (NL: 28% (26%–31%); BE: 28% (25%–30%); *p* = .079) (Supplementary Figure 2F).

**FIGURE 3:**
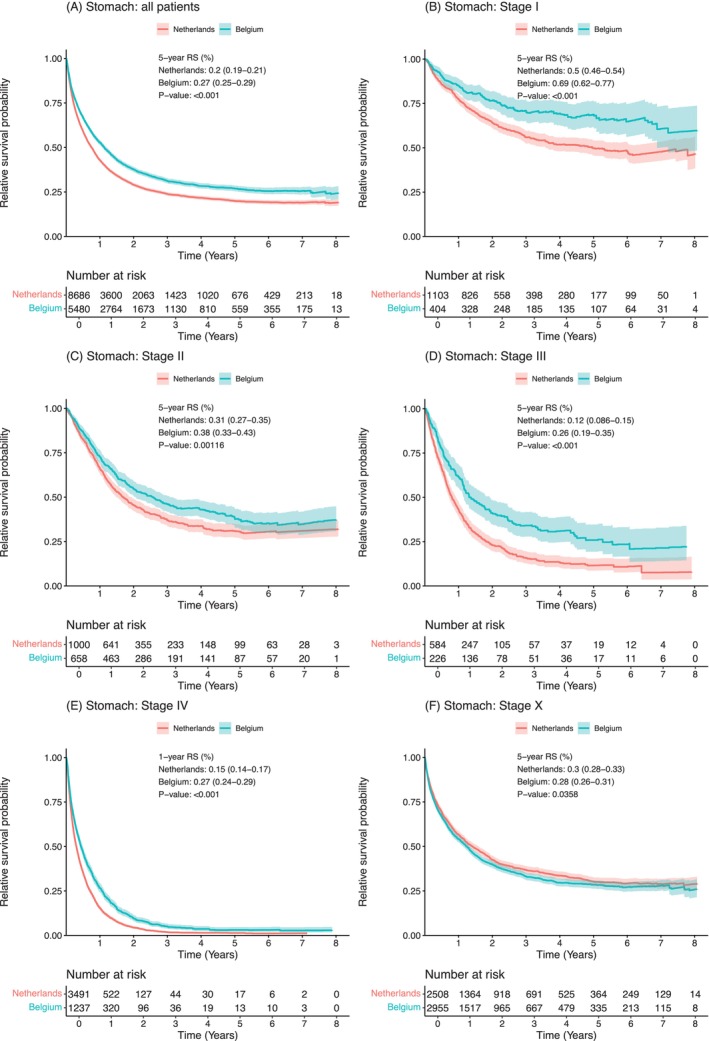
Relative survival probability curves for all patients with gastric cancer (A), and for patients with stage I (B), stage II (C), stage III (D), stage IV (E), and stage X (F). Relative survival was adjusted for baseline population mortality conditional on sex, year of birth, and country. *p*‐value denotes a log‐rank type test.

Patients from the Netherlands with gastric cancer had a significantly lower 5‐year relative survival compared to patients from Belgium (5‐year RS: NL: 20% (19%–21%); BE: 27% (25%–29%); *p* <.001) (Figure 3A). Across all known tumor stages, survival for patients from the Netherlands was lower than for patients from Belgium: stage I (5‐year RS: NL: 50% (46%–54%); BE: 69% (62%–77%); *p* <.001), stage II (5‐year RS: NL: 31% (27%–35%); BE: 38% (33%–43%); *p* = .001), stage III (5‐year RS: NL: 12% (0.09%–15%); BE: 26% (19%–35%); *p* <.001), stage IV (1‐year RS: NL: 15% (14%–17%); BE: 27% (24%–29%); *p* <.001) (Figure 3B–E). In contrast, patients with gastric cancer in an unknown stage from the Netherlands had a higher survival (5‐year RS: NL: 30% (28%–33%); BE: 28% (26%–31%); *p* = .036) (Figure 3F).

### 3.4 Excess hazard

The excess hazard baseline function up to 8 years since diagnosis was modeled with B‐splines of degree 3. For esophageal cancer all models used 2 knots, at 0.5 and 4 years, except for stage IV for which one knot at 2 years sufficed. For gastric cancer, most models used 2 knots at 0.4 and 1 year, except for stage I which needed an additional knot at 3 years to prevent an upward bent of the spline beyond 5 years.

**FIGURE 4:**
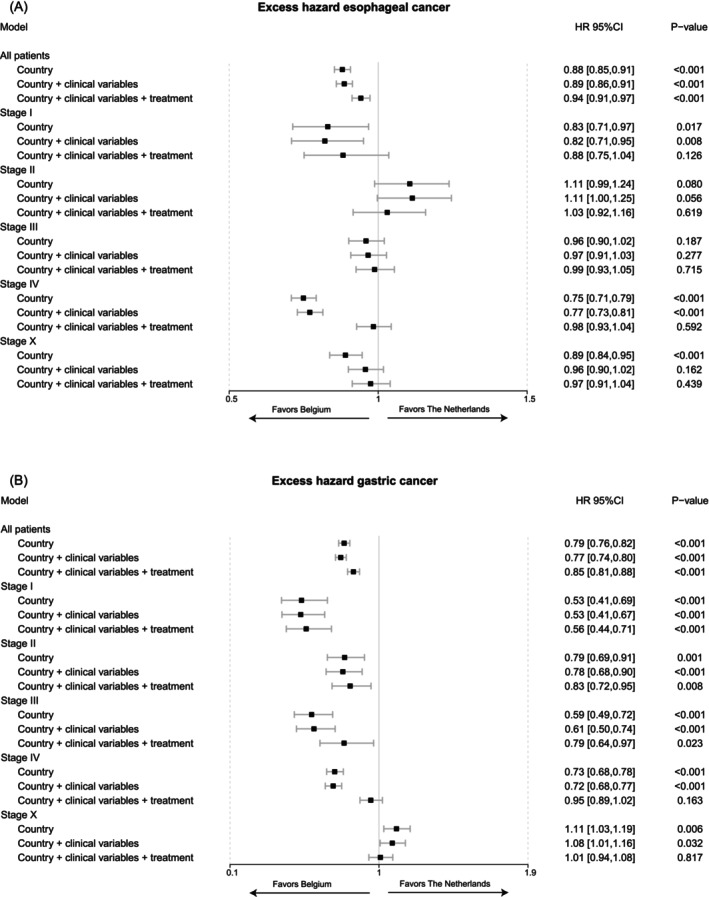
Forest plot depicting excess hazard ratios for (A) esophageal cancer and (B) gastric cancer. The sequential models first included country with the Netherlands as a reference, then country and clinical variables (EC: Sex, age, histology; GC: Sex, age) and lastly country, clinical variables, and the treatment categories as defined in Figure 1.

For all patients with esophageal cancer, the excess hazard ratio favored Belgium when controlled for sex, age, histology and treatment. However, while the excess mortality rate was 12% lower in Belgium without correction (HR 0.88, 95% CI [0.85–0.91]), this was only 6% when also correcting for treatment. (0.94 [0.91–0.97]) (Figure 4A). When analyzing stages separately, the excess hazard ratio favored Belgium for patients with stage I disease when controlled for clinical variables (0.82 [0.71–0.95]) and shifted slightly toward not favoring any country when also correcting for treatment (0.88 [0.75–1.04]). For patients with stage II disease, the excess mortality rate was 11% lower in the Netherlands without correction of any variables, which decreased to 3% when correcting for treatment. The confidence interval of both stages I and II was relatively large as these stage groups are the smallest subgroups. In stage III, there was no difference in excess mortality rates. For patients with stage IV disease, the excess hazard favored Belgium when controlled for clinical variables (0.77 [0.73–0.82]) but not when also correcting for treatment (0.98 [0.93–1.04]). For stage X, the excess hazard ratio favored Belgium (0.89 [0.84–0.95]) but not when correcting for clinical variables, including histology (0.96 [0.90–1.02]). For all patients with gastric cancer, the excess hazard ratio favored Belgium when correcting for both clinical variables and treatment. However, while the excess mortality rate was 21% lower in Belgium without any correction (0.79[0.76–0.82]), this was 15% lower when correcting for treatment (0.85 [0.81–0.88]) (Figure 4B). When analyzing stages separately, the excess hazard ratio also favored Belgium in stages I, II and III with correction for both clinical variables and treatment (Stage I: 0.56 [0.44–0.71]; Stage II: 0.83 [0.72–0.95]; Stage III: 0.79 [0.64–0.97]). For stage III, however, the excess mortality rate without correction in Belgium was 41% lower (0.59 [0.49–0.72]) compared to 21% when also correcting for treatment (0.79 [0.64–0.97]). In stage IV, the excess hazard ratio favored Belgium when correcting for clinical variables (Stage IV: 0.72 [0.68–0.77]) but not when also correcting for treatment (0.95 [0.89–1.02]). In stage X, the excess hazard ratio favored the Netherlands when correcting for clinical variables (1.08 [1.01–1.16]) but not when also correcting for treatment (1.01 [0.94–1.08]).

## 4 DISCUSSION

This study described notable differences in treatment and relative survival of patients with esophageal and gastric cancer between the Netherlands and Belgium. Survival was lower in the Netherlands for patients with gastric cancer in any stage and for patients with stage I and IV esophageal cancer. For patients with stage IV disease, the differences in relative survival appeared to be largely explained by differences in treatment. Treatment only partly mediated a difference in survival between the Netherlands and Belgium for patients with stage I and stage II esophageal cancer, and stage III gastric cancer. Despite correction for clinical variables and treatment, survival of patients with potentially curable gastric cancer remained lower in the Netherlands.

Relative survival was lower for patients from the Netherlands with both esophageal and gastric cancer in stage IV, but these differences disappeared when correcting for treatment. Patients with stage IV disease in Belgium received more local radical treatment such as chemoradiotherapy not followed by resection and surgical resection with (neo)adjuvant chemotherapy. In addition, patients were less likely to be treated with systemic therapy in the Netherlands. A similar low frequency of treatment with systemic therapy in the Netherlands compared to Belgium was previously observed in patients with malignant mesothelioma.^14^ In the Netherlands, the lower frequency of systemic therapy was accompanied by a higher frequency of best supportive care for both gastric and esophageal metastatic disease. Best supportive care includes “radiotherapy only” as this does not improve survival and is generally used for relieving dysphagia in esophageal cancer.^15^, ^16^, ^17^ The high frequency of treatment with systemic therapy could account for the observed better survival of patients with stage IV disease in Belgium, as systemic therapy has been shown to improve survival compared to best supportive care for patients with gastric and esophageal cancer.^18^, ^19^, ^20^, ^21^

In the Netherlands, significant emphasis is placed on “appropriate care” which aims to deliver effective patient care by avoiding unnecessary interventions, particularly in case cure cannot be achieved. This approach seeks to minimize treatments that offer limited benefits and pose potential side effects that affect the quality of life of the patients, while also considering the financial implications for society.^22^ As part of this approach, end‐of‐life discussions occur relatively more frequently in the Netherlands compared to for example Belgium.^23^ These end‐of‐life discussions are associated with a choice for less invasive treatment, including less palliative systemic therapy.^24^ However, given the observed differences in relative survival between the Netherlands and Belgium, the question arises whether the Dutch caution in prescribing palliative systemic therapy is unduly restrictive. Unfortunately, in our study we do not have data available on differences in quality of life between patients from the Netherlands and Belgium. However, two systematic reviews demonstrated that quality of life remains stable during an extended period of time during chemotherapy for metastatic esophagogastric cancer compared to best supportive care.^25^, ^26^ Real‐world data from the Netherlands has actually shown an improvement in quality of life during chemotherapy, while quality of life deteriorated at disease progression irrespective of treatment with chemotherapy.^27^ Recent treatment advances with the addition of nivolumab to chemotherapy in the first line have shown stable or even improved health‐related quality of life, with a lower chance of definitive deterioration.^28^ Also, importantly, real‐world data indicate that patients treated at Dutch centers with a high volume of systemic therapy prescriptions experience better survival outcomes, while these centers administer less systemic therapy in the last 3 months of life.^29^, ^30^, ^31^ This observation suggests that these centers are proficient in selecting patients who are most likely to derive significant survival benefits from chemotherapy. Taken together, the reported survival benefits of systemic therapy and its minimal negative impact on quality of life challenge the Dutch reserve regarding the prescription of palliative systemic therapy in metastatic gastric and esophageal cancer.

Relative survival was higher for patients with stage I esophageal cancer in Belgium, a difference that decreased when correcting for treatment. We observed that patients with stage I disease in Belgium were more frequently treated with surgery only, whereas in the Netherlands these patients were also treated with neoadjuvant chemoradiotherapy followed by resection. The different treatment regimens can possibly be explained by a difference in the incidence of T1N0 and T2N0 tumors, which together make up stage I tumors. In Belgium, proportionally more T1N0 tumors were diagnosed, for which the standard of care is endoscopic resection or surgical resection only, whereas in the Netherlands more T2N0 tumors were diagnosed.^32^ The standard of care for this subgroup is controversial, with evidence for and against neoadjuvant treatment in addition to surgery. It seems that in the Netherlands neoadjuvant treatment for this subgroup is frequently prescribed. Literature suggests that T2 tumors have a worse prognosis than T1 tumors.^33^, ^34^ This may be an explanation for the poorer survival of patients with stage I esophageal cancer in the Netherlands. Alternatively, patients with T2N0 could experience an increase in postoperative mortality after treatment with neoadjuvant chemoradiotherapy, as suggested by Marriette et al.^35^ It is important to note that differences in treatment do not fully mediate the difference in survival we found. There was still a decrease of excess mortality of 12% in Belgium compared to the Netherlands after correcting for treatment. The larger confidence intervals, likely a result of the smaller subgroup of stage I patients, make it harder to draw definitive conclusions.

The Dutch and Belgian healthcare systems, while both highly regarded, exhibit distinct differences in structure, operation, accessibility, and specialist referral processes. Accessibility in the Netherlands requires patients to first see a general practitioner (GP) who acts as a gatekeeper and provides referrals to specialists if needed.^36^ In contrast, accessibility in Belgium is more direct, as patients have the freedom to consult specialists without a referral from a GP. Possibly, this fast access may result in less diagnostic delay in Belgium, which has been associated with a lower disease stage and so could serve as a potential explanation for the higher frequency of T1 tumors observed.^37^, ^38^, ^39^ It is noteworthy that centralized surgical care in high‐volume specialized centers was implemented for esophagus surgery in July 2019 in Belgium compared to 2013 in the Netherlands.^40^, ^41^ This makes the higher survival in Belgium of curable disease even more striking.

Survival of patients with gastric cancer was higher in Belgium across curable stages I, and II irrespective of clinical characteristics and treatment. For stage III, treatment only partly mediated the difference in survival between the two countries. A similar result was found in an article investigating overall survival in multiple countries over time.^5^ Here correction for surgery did not improve overall survival in the Netherlands and Belgium after 2010. Therefore, the factor that mediated the higher relative survival in Belgium compared to the Netherlands remains unknown. One possible explanation is a tumor‐intrinsic factor causing patients in the Netherlands to be more resistant to treatment. Recent research has shown that the proportion of diffuse subtype of gastric cancer is increasing in the Netherlands.^42^, ^43^ Survival for this subtype is poor, and has not improved much in recent years despite treatment advances. Currently, it is unknown what the incidence is of this subgroup in Belgium.

In the context of this study, it is important to consider that a large number of patients from Belgium had an unknown tumor stage (X) compared to patients from the Netherlands. Almost 33% of the Belgian patients with esophageal cancer and 42% for gastric cancer had an unknown cM category. This is most likely the result of differences in the method of cancer registration and incomplete data delivery of the T, N, and M categories to the BCR. In the stage X groups, survival was lower for patients with gastric cancer in Belgium, but higher among patients with esophageal cancer. Had the tumor stage been known, it would likely not have changed outcomes for the other staging subgroups of patients from the Netherlands, as only a very small fraction was unknown. For Belgian patients, however, outcomes might have changed due to the relatively large number of unknowns. For Belgian patients with esophageal and gastric cancer with stage X, 5‐year relative survival corresponded roughly with Stage III. This might indicate that the survival of potentially curable clinical stage was overestimated whilst the survival of palliative clinical stage was underestimated by the exclusion of the stage X patients. It also calls into question whether the diagnostic approach to staging in the Netherlands and Belgium can be compared accurately, which is important to consider for our results per stage subgroup.

This study is the first population‐based direct comparison between the Netherlands and Belgium regarding survival and treatment using real‐world data of patients with esophageal and gastric cancer. The relative survival and excess hazard modeling allowed us to investigate survival differences and identify potential mediators while correcting for baseline population mortality, conditional on sex, age, and country. A limitation lies in a possible interaction between country and treatment as the identified mediator. Although both countries uphold the European guidelines for treating patients with esophageal and gastric cancer,^16^, ^32^, ^44^ small differences between countries may still arise in selecting patients that are fit for treatment, in average time to start of treatment, or in treatment protocols and preferred surgical procedures. These differences could result in biased estimates of the survival differences found. Another limitation of this study lies in the limited number of clinical variables available for correction in the excess hazard models such as smoking, performance status or her2neu positivity between countries. These factors are known to impact survival of patients with esophageal and gastric cancer or of the general population.^45^ The proportion of patients with esophageal squamous cell carcinoma, a notoriously smoking‐related cancer, was higher in Belgium. Indeed, the percentage of smokers in Belgium is approximately 1% higher than in the Netherlands.^46^ Smoking status influences the expected mortality rate, and therefore the survival of a population with a higher proportion of smokers is lower than the survival of the general population.^47^ This could mean that the higher relative survival for patients in Belgium is underestimated compared to patients in the Netherlands. However, it has been reported that the difference between overall survival and cancer‐specific survival in esophageal cancer is very small,^48^ which disputes the impact of smoking status on survival in patients with esophageal cancer. In any case, future studies should aim to expand on clinically relevant factors to investigate survival discrepancies.

In conclusion, survival of patients with esophageal cancer and gastric cancer differs between the Netherlands and Belgium. For patients with stage IV disease, the difference in survival can most likely be attributed to differences in treatment. Although treatment explains part of the survival differences for patients with curable disease, other causes remain largely unknown.

## AUTHOR CONTRIBUTIONS

**Benthe H. Doeve:** Investigation; writing – original draft; writing – review and editing; visualization; project administration; methodology. **Steven C. Kuijper:** Methodology; visualization; writing – original draft; writing – review and editing; formal analysis; data curation. **Geert Silversmit:** Writing – review and editing; methodology; formal analysis; funding acquisition; conceptualization. **Lien van Walle:** Writing – review and editing; funding acquisition; formal analysis; conceptualization. **Philippe Nafteux:** Writing – review and editing. **Camiel Rosman:** Writing – review and editing. **Pauline A. J. Vissers:** Methodology; writing – review and editing. **Paul Jeene:** Writing – review and editing. **Laurens V. Beerepoot:** Writing – review and editing. **Sarah Derks:** Writing – review and editing. **Amine Karimi:** Writing – review and editing. **Maarten F. Bijlsma:** Writing – review and editing. **Hanneke W. M. van Laarhoven:** Conceptualization; writing – review and editing. **Rob H. A. Verhoeven:** Conceptualization; writing – review and editing; methodology; formal analysis; data curation; supervision.

## FUNDING INFORMATION

The research was supported by the Foundation Against Cancer, Brussels, Belgium (Stichting tegen Kanker, reference no. 2023‐003).

## CONFLICT OF INTEREST STATEMENT

C.R has received research grants from Johnson&Johnson, Medtronic, and ZonMw, and acted as a consultant for DEKRA medical BV. L.V.B. reports speaker roles for Servier, BMS, Congress Care, Ipsen, Medtalks, Benecke and Travel Congress Management. S.D. reports a consultant or advisory role for BMS (related to checkpoint inhibitors); research funding, medication supply, or both from Incyte (related to checkpoint inhibitors); and speaker roles for Servier, BMS, and Benecke. M.F.B. has received research funding from Celgene, Frame Therapeutics, and Lead Pharma, and has acted as a consultant to Servier and Olympus. H.W.M.vL. Consultant or advisory role: Amphera, Anocca, Astellas, AstraZeneca, Beigene, BMS, Boehringer, Daiichi, Dragonfly, Eli Lilly, MSD, Myeloid, Nordic Pharma, Servier. Research funding and/or medication supply: Auristone, Bayer, BMS, Celgene, Janssen, Incyte, Eli Lilly, MSD, Merck, ORCA, Nordic Pharma, Philips, Roche, Servier. Speaker role: Astellas, Beigene, Benecke, BMS, Daiichi‐Sankyo, JAAP, Medtalks, Novartis, Springer, Travel Congress Management B.V. R.H.A.V has received research grants from Bristol‐Myers Squibb and Roche and reports consultancy for Daiichi Sankyo. None of these parties were involved in the design of this study or drafting of the manuscript. All other authors have no competing interests to declare.

## ETHICS STATEMENT

This observational study does not require approval from an Ethical Committee in Belgium or the Netherlands. For Belgium, the study was performed within the legal and ethical framework of the Belgian Cancer Registry (BCR). Based on the Coordinated law of 10 May 2015 (art. 138) (Het Belgisch Staatsblad. [fgov.be]; https://www.ejustice.just.fgov.be/doc/rech_n.htm), the BCR has a legal task to collect data on cancer, subject it to quality control, process and analyze it, encrypt and store it, and report on it. Its activities are monitored and evaluated by the Advisory Committee of Users, an independent committee whose duly qualified members are appointed by law. In the Netherlands, no approval from an ethics committee is needed according to the Central Committee on Research Involving Human Subjects (CCMO). Use of the anonymous data for our study was approved by the Privacy Review Board of the Netherlands Cancer Registry and the Biobank and Data Access Committee of the Princess Máxima Center for Pediatric Oncology, following the principles of the Code of Good Conduct of the Federa (https://www.federa.org/codes-conduct).

## Supporting information


**Supplementary Table 1** Number and proportion of clinical T‐stage and clinical N‐stage per clinical stage for esophageal cancer.
**Supplementary Table 2**. Number and proportion of types of treatment per clinical stage for esophageal cancer.
**Supplementary Table 3**. Number and proportion of types of treatment per clinical stage for gastric cancer.
**Supplementary Figure 1**. Distribution of treatment categories per clinical stage for patients with esophageal cancer (A) and gastric cancer (B) in the Netherlands and Belgium. For the Netherlands, Best Supportive Care was differentiated from other or unknown. CRT = chemoradiotherapy, nCRT = neoadjuvant chemoradiotherapy.
**Supplementary Figure 2**. Relative survival probability curves for all patients with esophageal adenocarcinoma (A), and for patients with stage I (B), stage II (C), stage III (D), stage IV (E), and stage X (F) disease. Relative survival was adjusted for baseline population mortality conditional on sex, year of birth, and country. *P*‐value denotes a log‐rank‐type test. AC = adenocarcinoma.
**Supplementary Figure 3**. Relative survival probability curves for all patients with esophageal squamous cell carcinoma (A), and for patients with stage I (B), stage II (C), stage III (D), stage IV (E), and stage X (F) disease. Relative survival was adjusted for baseline population mortality conditional on sex, year of birth, and country. *P*‐value denotes a log‐rank‐type test. SCC = squamous cell carcinoma.

## Data Availability

The data that support the findings of this study are available from the corresponding author upon reasonable request.
