# Supplementary material for: Treatment and survival of patients with esophageal and gastric cancer in the Netherlands and Belgium: A population‐based comparison
Source: Int J Cancer. 2025 Nov 19;158(7):1868–80. doi: 10.1002/ijc.70225 (PMC12875157; doi:10.1002/ijc.70225)
Supplement: Supplementary file 1 — Supplementary Table 1 Number and proportion of clinical T‐stage and clinical N‐stage per clinical stage for esophageal cancer. Supplementary Table 2. Number and proportion of types of treatment per clinical stage for esophageal cancer. Supplementary Table 3. Number and proportion of types of treatment per clinical stage for gastric cancer. Supplementary Figure 1. Distribution of treatment categories per clinical stage for patients with esophageal cancer (A) and gastric cancer (B) in the Netherlands and Belgium. For the Netherlands, Best Supportive Care was differentiated from other or unknown. CRT = chemoradiotherapy, nCRT = neoadjuvant chemoradiotherapy. Supplementary Figure 2. Relative survival probability curves for all patients with esophageal adenocarcinoma (A), and for patients with stage I (B), stage II (C), stage III (D), stage IV (E), and stage X (F) disease. Relative survival was adjusted for baseline population mortality conditional on sex, year of birth, and country. P‐value denotes a log‐rank‐type test. AC = adenocarcinoma. Supplementary Figure 3. Relative survival probability curves for all patients with esophageal squamous cell carcinoma (A), and for patients with stage I (B), stage II (C), stage III (D), stage IV (E), and stage X (F) disease. Relative survival was adjusted for baseline population mortality conditional on sex, year of birth, and country. P‐value denotes a log‐rank‐type test. SCC = squamous cell carcinoma. [file IJC-158-1868-s001.pdf]

Treatment and survival of patients with esophageal and gastric cancer in the Netherlands and Belgium: a population-based comparison

Benthe H. Doeve, Steven C. Kuijper, Geert Silversmit, Lien van Walle, Philippe Nafteux, Camiel Rosman, Pauline A. J. Vissers, Paul Jeene, Laurens Beerepoot, Sarah Derks, Amine Karimi, Maarten F. Bijlsma, Hanneke W.M. van Laarhoven, Rob H.A. Verhoeven

Table of content

Supplementary table 1 ……………………………………2
Supplementary table 2 ……………………………………4
Supplementary table 3 ……………………………………5
Supplementary figure 1 ……………………………………6
Supplementary figure 2 ……………………………………7
Supplementary figure 3 ……………………………………8

Supplementary table 1 Number and proportion of clinical T-stage and clinical N-stage per clinical stage for esophageal cancer.

	Stage I		Stage II		Stage III		Stage IV		Stage X	
	Netherlands	Belgium	Netherlands	Belgium	Netherlands	Belgium	Netherlands	Belgium	Netherlands	Belgium
T1 N0	526 (27.3)	381 (50.1)	-	-	-	-	22 (0.4)	14 (0.7)	-	44 (1.1)
T1 N1	-	-	64 (2.7)	83 (9.7)	-	-	20 (0.3)	17 (0.9)	-	20 (0.5)
T1 N2	-	-	-	-	13 (0.2)	15 (0.7)	16 (0.3)	13 (0.7)	-	3 (0.1)
T1 N3	-	-	-	-	4 (0.1)	14 (0.6)	-	-	-	-
T1 Nx	-	-	-	-	-	-	7 (0.1)	13 (0.7)	127 (4.4)	110 (2.8)
T2 N0	1400 (72.7)	379 (49.9)	-	-	-	-	227 (3.9)	25 (1.3)	-	51 (1.3)
T2 N1	-	-	861 (36.7)	315 (36.9)	-	-	597 (10.2)	80 (4.3)	-	66 (1.7)
T2 N2	-	-	-	-	336 (6.3)	78 (3.5)	605 (10.3)	27 (1.4)	-	4 (0.1)
T2 N3	-	-	-	-	180 (3.4)	32 (1.5)	-	-	-	-
T2 Nx	-	-	-	-	-	-	112 (1.9)	20 (1.1)	107 (3.7)	115 (2.9)
T3 N0	-	-	1423 (60.6)	455 (53.3)	-	-	130 (2.2)	44 (2.3)	-	50 (1.3)
T3 N1	-	-	-	-	2262 (42.7)	1143 (52.0)	583 (9.9)	385 (20.6)	-	257 (6.5)
T3 N2	-	-	-	-	1139 (21.5)	404 (18.4)	670 (11.4)	222 (11.9)	-	66 (1.7)
T3 N3	-	-	-	-	412 (7.8)	229 (10.4)	-	-	-	-
T3 Nx	-	-	-	-	-	-	36 (0.6)	65 (3.5)	104 (3.6)	248 (6.3)
T4 N0	-	-	-	-	147 (2.8)	45 (2.0)	45 (0.8)	7 (0.4)	-	5 (0.1)
T4 N1	-	-	-	-	261 (4.9)	109 (5.0)	155 (2.6)	65 (3.5)	1 (0.0)	26 (0.7)
T4 N2	-	-	-	-	203 (3.8)	43 (2.0)	199 (3.4)	67 (3.6)	-	15 (0.4)
T4 N3	-	-	-	-	118 (2.2)	39 (1.8)	-	-	-	-
T4 Nx	-	-	-	-	-	-	35 (0.6)	37 (2.0)	52 (1.8)	64 (1.6)
Tx N0	-	-	-	-	-	-	395 (6.7)	49 (2.6)	931 (32.6)	227 (5.8)
Tx N1	-	-	-	-	-	-	836 (14.2)	180 (9.6)	391 (13.7)	160 (4.1)
Tx N2	-	-	-	-	-	-	778 (13.3)	67 (3.6)	163 (5.7)	37 (0.9)
Tx N3	-	-	-	-	221 (4.2)	49 (2.2)	-	-	-	-
Tx Nx	-	-	-	-	-	-	402 (6.8)	476 (25.4)	978 (34.3)	2363 (60.1)

	Stage I		Stage II		Stage III		Stage IV		Stage X	
	Netherlands	Belgium	Netherlands	Belgium	Netherlands	Netherlands	Belgium	Netherlands	Belgium	Netherlands
T1 N0	223 (20.2)	160 (39.6)	-	-	-	-	10 (0.3)	10 (0.8)	-	21 (0.7)
T1 N1	13 (1.2)	20 (5.0)	-	-	-	-	14 (0.4)	6 (0.5)	-	7 (0.2)
T1 N2	-	-	-	3 (0.5)	-	-	9 (0.3)	6 (0.5)	-	-
T1 N3	-	-	1 (0.1)	-	-	-	1 (0.0)	6 (0.5)	-	-
T1 Nx	-	-	-	-	-	-	4 (0.1)	17 (1.4)	46 (1.8)	62 (2.1)
T2 N0	867 (78.6)	224 (55.4)	-	-	-	-	229 (6.6)	13 (1.1)	-	45 (1.5)
T2 N1	-	-	244 (24.4)	75 (11.4)	-	-	219 (6.3)	21 (1.7)	-	22 (0.7)
T2 N2	-	-	109 (10.9)	18 (2.7)	-	-	218 (6.2)	11 (0.9)	-	5 (0.2)
T2 N3	-	-	-	-	11 (1.9)	5 (2.2)	29 (0.8)	6 (0.5)	-	-
T2 Nx	-	-	-	-	-	-	123 (3.5)	20 (1.6)	95 (3.8)	62 (2.1)
T3 N0	-	-	345 (34.5)	224 (34.0)	-	-	105 (3.0)	36 (2.9)	-	36 (1.2)
T3 N1	-	-	245 (24.5)	311 (47.3)	-	-	141 (4.0)	128 (10.3)	-	79 (2.7)
T3 N2	-	-	-	-	147 (25.2)	101 (44.7)	171 (4.9)	66 (5.3)	-	30 (1.0)
T3 N3	-	-	-	-	18 (3.1)	31 (13.7)	22 (0.6)	33 (2.7)	-	5 (0.2)
T3 Nx	-	-	-	-	-	-	49 (1.4)	44 (3.6)	50 (2.0)	99 (3.4)
T4A N0	-	-	56 (5.6)	27 (4.1)	-	-	37 (1.1)	20 (1.6)	-	9 (0.3)
T4A N1	-	-	-	-	41 (7.0)	48 (21.2)	41 (1.2)	60 (4.9)	-	17 (0.6)
T4A N2	-	-	-	-	30 (5.1)	21 (9.3)	37 (1.1)	34 (2.7)	-	13 (0.4)
T4A N3	-	-	-	-	6 (1.0)	4 (1.8)	14 (0.4)	23 (1.9)	-	4 (0.1)
T4A Nx	-	-	-	-	-	-	24 (0.7)	32 (2.6)	17 (0.7)	32 (1.1)
T4B N0	-	-	-	-	156 (26.7)	2 (0.9)	91 (2.6)	6 (0.5)	-	2 (0.1)
T4B N1	-	-	-	-	108 (18.5)	7 (3.1)	110 (3.2)	4 (0.3)	-	2 (0.1)
T4B N2	-	-	-	-	65 (11.1)	6 (2.7)	95 (2.7)	6 (0.5)	-	-
T4B N3	-	-	-	-	2 (0.3)	1 (0.4)	24 (0.7)	8 (0.6)	-	-
T4B Nx	-	-	-	-	-	-	94 (2.7)	5 (0.4)	49 (2.0)	6 (0.2)
Tx N0	-	-	-	-	-	-	410 (11.7)	72 (5.8)	1229 (49.0)	258 (8.7)
Tx N1	-	-	-	-	-	-	350 (10.0)	97 (7.8)	246 (9.8)	88 (3.0)
Tx N2	-	-	-	-	-	-	304 (8.7)	32 (2.6)	124 (4.9)	19 (0.6)
Tx N3	-	-	-	-	-	-	40 (1.1)	17 (1.4)	4 (0.2)	2 (0.1)
Tx Nx	-	-	-	-	-	-	476 (13.6)	398 (32.2)	648 (25.8)	2030 (68.7)

Supplementary table 2 Number and proportion of types of treatment per clinical stage for esophageal cancer

	Esophageal cancer									
	I		II		III		IV		X	
	Netherlands	Belgium	Netherlands	Belgium	Netherlands	Belgium	Netherlands	Belgium	Netherlands	Belgium
N	1926	760	2348	853	5296	2200	5870	1873	2854	3931
Chemotherapy only	18 (0.9)	18 (2.4)	39 (1.7)	42 (4.9)	466 (8.8)	271 (12.3)	1950 (33.2)	929 (49.6)	79 (2.8)	540 (13.7)
CRT not followed by resection	275 (14.3)	94 (12.4)	459 (19.5)	192 (22.5)	1111 (21.0)	599 (27.2)	365 (6.2)	241 (12.9)	256 (9.0)	651 (16.6)
Endoscopic resection	248 (12.9)	108 (14.2)	6 (0.3)	19 (2.2)	3 (0.1)	64 (2.9)	1 (0.0)	72 (3.8)	404 (14.2)	506 (12.9)
Neoadjuvant chemoradiotherapy followed by surgery with(out) adjuvant therapy	642 (33.3)	20 (2.6)	1240 (52.8)	205 (24.0)	2054 (38.8)	541 (24.6)	47 (0.8)	19 (1.0)	245 (8.6)	186 (4.7)
Other or unknown	201 (10.4)	60 (7.9)	165 (7.0)	65 (7.6)	630 (11.9)	204 (9.3)	2005 (34.2)	498 (26.6)	1201 (42.1)	1089 (27.7)
Radiotherapy only	219 (11.4)	49 (6.4)	219 (9.3)	44 (5.2)	637 (12.0)	41 (1.9)	1455 (24.8)	45 (2.4)	459 (16.1)	118 (3.0)
Surgical resection only	258 (13.4)	298 (39.2)	91 (3.9)	134 (15.7)	94 (1.8)	123 (5.6)	4 (0.1)	19 (1.0)	115 (4.0)	444 (11.3)
(Neo)adjuvant chemotherapy with surgery	65 (3.4)	113 (14.9)	129 (5.5)	152 (17.8)	301 (5.7)	357 (16.2)	43 (0.7)	50 (2.7)	95 (3.3)	397 (10.1)

Supplementary table 3 Number and proportion of types of treatment per clinical stage for gastric cancer

	Gastric cancer									
	I		II		III		IV		X	
	Netherlands	Belgium	Netherlands	Belgium	Netherlands	Belgium	Netherlands	Belgium	Netherlands	Belgium
N	1103	404	1000	658	584	226	3491	1237	2508	2955
Chemotherapy only	51 (4.6)	7 (1.7)	86 (8.6)	76 (11.6)	87 (14.9)	49 (21.7)	1229 (35.2)	660 (53.4)	107 (4.3)	334 (11.3)
Endoscopic resection	42 (3.8)	18 (4.5)	2 (0.2)	7 (1.1)	0 (0.0)	2 (0.9)	1 (0.0)	26 (2.1)	58 (2.3)	115 (3.9)
Other or unknown	197 (17.9)	65 (16.1)	201 (20.1)	71 (10.8)	232 (39.7)	28 (12.4)	2081 (59.6)	454 (36.7)	998 (39.8)	1061 (35.9)
Surgical resection only	411 (37.3)	200 (49.5)	246 (24.6)	168 (25.5)	99 (17.0)	33 (14.6)	105 (3.0)	38 (3.1)	716 (28.5)	811 (27.4)
(Neo)adjuvant chemotherapy with surgery	402 (36.4)	114 (28.2)	465 (46.5)	336 (51.1)	166 (28.4)	114 (50.4)	75 (2.1)	59 (4.8)	629 (25.1)	634 (21.5)

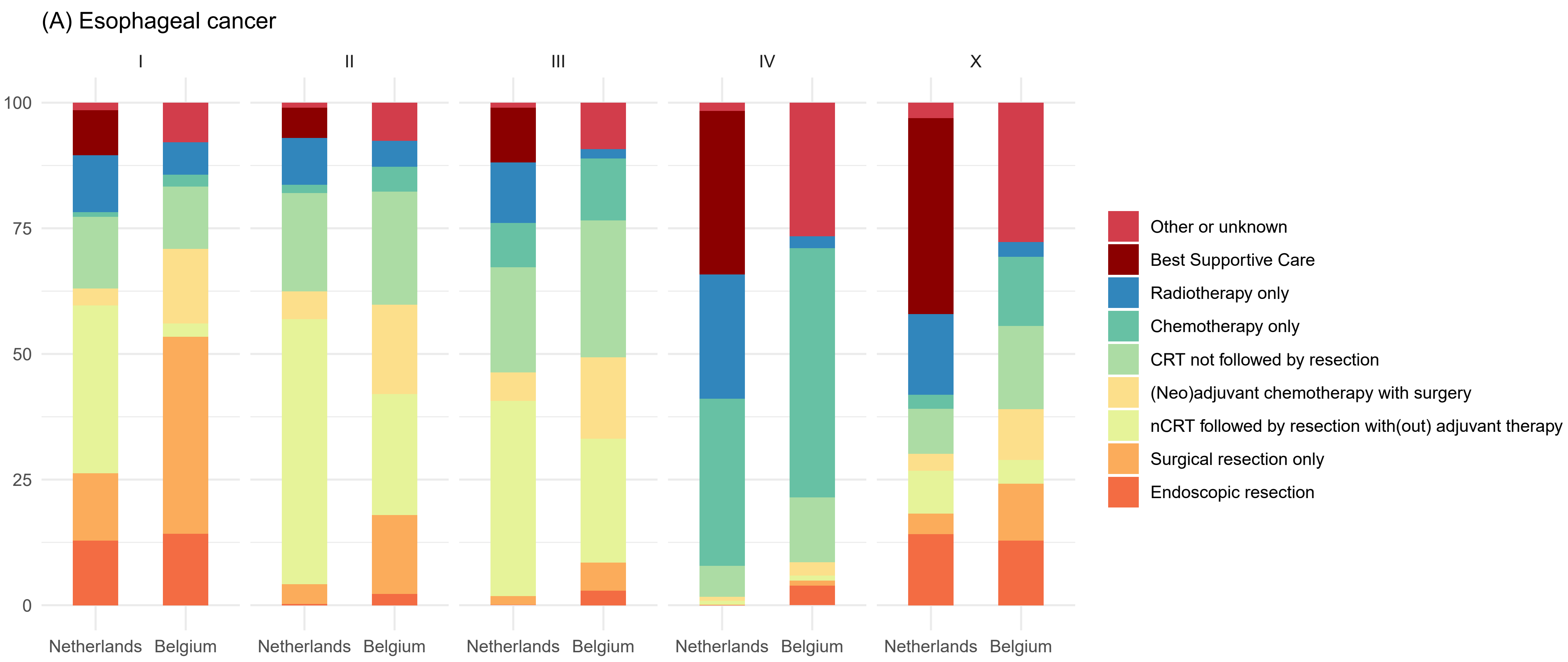

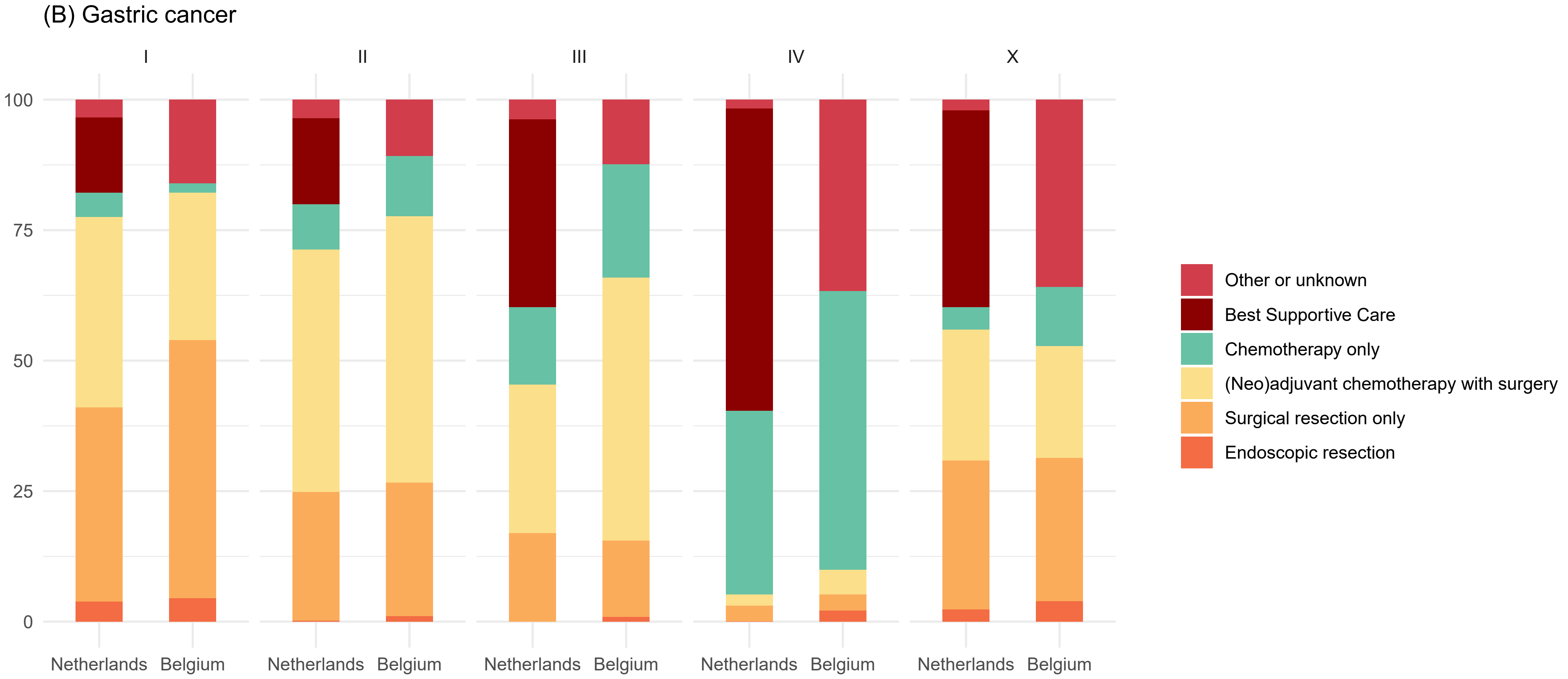

Supplementary Figure 1. Distribution of treatment categories per clinical stage for patients with esophageal cancer (A) and gastric cancer (B) in the Netherlands and Belgium. For the Netherlands, Best Supportive Care was differentiated from other or unknown. CRT = chemoradiotherapy, nCRT = neoadjuvant chemoradiotherapy.

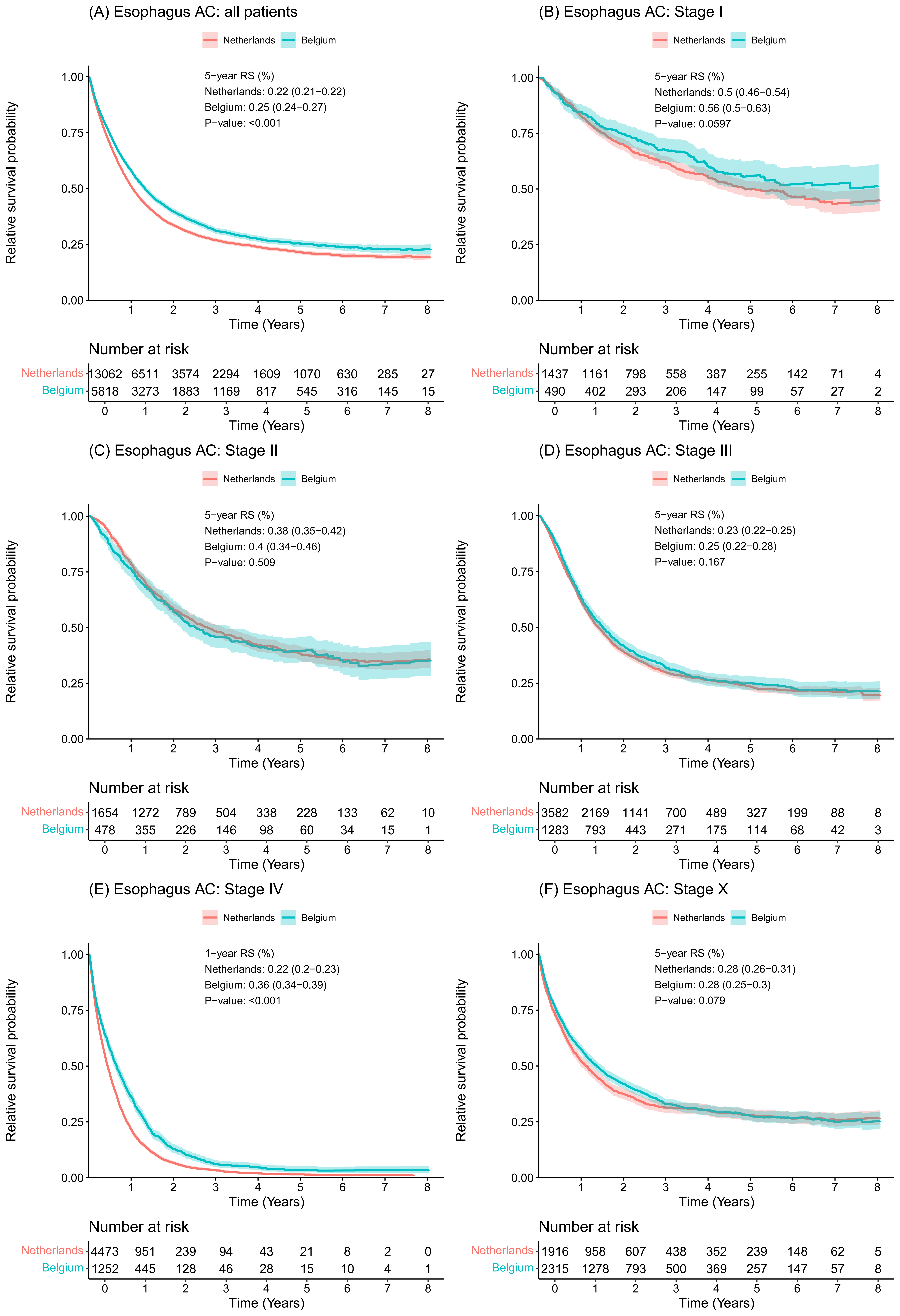

Supplementary Figure 2. Relative survival probability curves for all patients with esophageal adenocarcinoma (A), and for patients with stage I (B), stage II (C), stage III (D), stage IV (E), and stage X (F) disease. Relative survival was adjusted for baseline population mortality conditional on sex, year of birth, and country. P-value denotes a log-rank-type test. AC = adenocarcinoma.

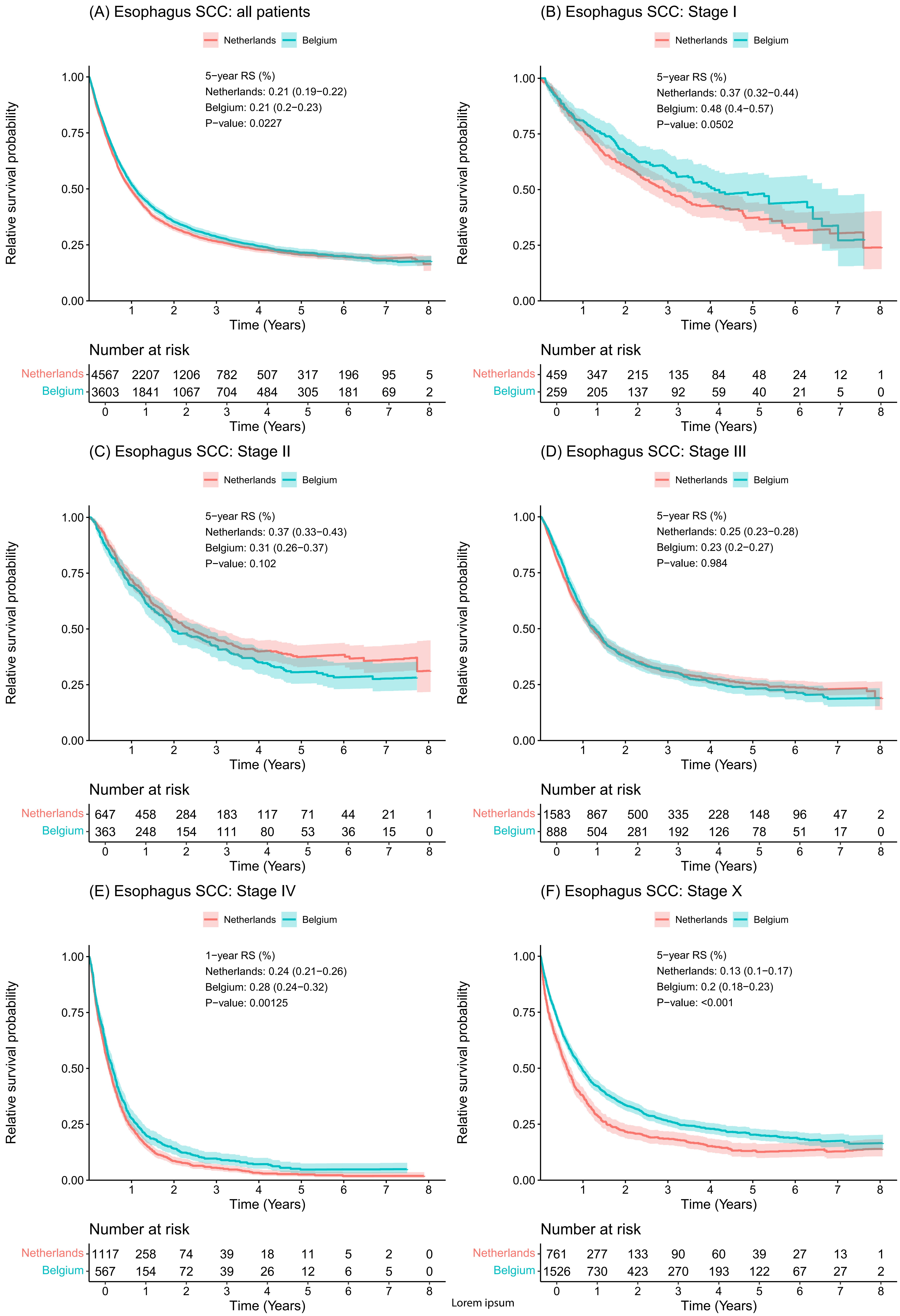

Supplementary Figure 3. Relative survival probability curves for all patients with esophageal squamous cell carcinoma (A), and for patients with stage I (B), stage II (C), stage III (D), stage IV (E), and stage X (F) disease. Relative survival was adjusted for baseline population mortality conditional on sex, year of birth, and country. P-value denotes a log-rank-type test. SCC = squamous cell carcinoma
